# Phylogenetic and Functional Diversity of Saprolegniales and Fungi Isolated from Temperate Lakes in Northeast Germany

**DOI:** 10.3390/jof7110968

**Published:** 2021-11-13

**Authors:** Hossein Masigol, Jason Nicholas Woodhouse, Pieter van West, Reza Mostowfizadeh-Ghalamfarsa, Keilor Rojas-Jimenez, Tobias Goldhammer, Seyed Akbar Khodaparast, Hans-Peter Grossart

**Affiliations:** 1 Experimental Limnology, Leibniz Institute for Freshwater Ecology and Inland Fisheries (IGB), 16775 Neuglobsow, Germany; hossein.masigol@gmail.com (H.M.); woodhouse@igb-berlin.de (J.N.W.); 2 Aberdeen Oomycete Laboratory, International Centre for Aquaculture Research and Development (ICARD), Institute of Medical Sciences, University of Aberdeen, Foresterhill, Aberdeen AB25 2ZD, UK; p.vanwest@abdn.ac.uk; 3 Department of Plant Protection, School of Agriculture, Shiraz University, Shiraz 71946-84334, Iran; rmostofi@shirazu.ac.ir; 4 Escuela de Biologia, Universidad de Costa Rica, San Jose 11501, Costa Rica; keilor.rojas@gmail.com; 5 Department of Ecohydrology and Biogeochemistry, Leibniz-Institute of Freshwater Ecology and Inland Fisheries, 12587 Berlin, Germany; goldhammer@igb-berlin.de; 6 Department of Plant Protection, Faculty of Agricultural Sciences, University of Guilan, Rasht 41996-13776, Iran; Blumeria2015@gmail.com; 7 Institute for Biochemistry and Biology, Potsdam University, 14469 Potsdam, Germany

**Keywords:** freshwater ecosystems, aquatic carbon cycling, plant litter degradation, humic substances production, *Saprolegnia*, *Achlya*, *Leptolegnia*, *Cladosporium*, *Penicillium*

## Abstract

The contribution of fungi to the degradation of plant litter and transformation of dissolved organic matter (humic substances, in particular) in freshwater ecosystems has received increasing attention recently. However, the role of Saprolegniales as one of the most common eukaryotic organisms is rarely studied. In this study, we isolated and phylogenetically placed 51 fungal and 62 Saprolegniales strains from 12 German lakes. We studied the cellulo-, lignino-, and chitinolytic activity of the strains using plate assays. Furthermore, we determined the capacity of 10 selected strains to utilize 95 different labile compounds, using Biolog FF MicroPlates™. Finally, the ability of three selected strains to utilize maltose and degrade/produce humic substances was measured. *Cladosporium* and *Penicillium* were amongst the most prevalent fungal strains, while *Saprolegnia*, *Achlya*, and *Leptolegnia* were the most frequent Saprolegniales strains. Although the isolated strains assigned to genera were phylogenetically similar, their enzymatic activity and physiological profiling were quite diverse. Our results indicate that Saprolegniales, in contrast to fungi, lack ligninolytic activity and are not involved in the production/transformation of humic substances. We hypothesize that Saprolegniales and fungi might have complementary roles in interacting with dissolved organic matter, which has ecological implications for carbon cycling in freshwater ecosystems.

## 1. Introduction

Dissolved organic matter (DOM) mainly constitutes a heterogeneous mixture of aliphatic and aromatic polymers, and thus plays a pivotal role in shaping aquatic ecosystems [1]. It consists of labile autochthonous DOM (DOM_auto_), generally produced by phytoplankton, and more refractory allochthonous DOM (DOM_allo_), which mainly originates from plant litter degradation [2]. The most significant fraction of DOM_allo_ (up to 80%) consists of humic substances (HS), which have been defined as a category of heterogeneous organic and high-molecular-weight matter [3]. Although HS are extremely resistant to biodegradation by heterotrophic bacteria, they are believed to be degraded by fungi with important consequences for aquatic carbon cycling [4]. The production of polymer-degrading enzymes [5] and reactive oxygen species (ROS) [6] facilitates the interaction between fungi and recalcitrant organic matter which eventually contributes to the aquatic microbial loop and transform or mineralize organic carbon [7]. 

However, while most studies have focused on the ability of terrestrial fungi to degrade/transform DOM (HS, in particular), it has not yet been established whether freshwater fungi contribute the same. In 1998, Claus and Filip [8] isolated a phenoloxidase-producing fungus named *Cladosporium cladosporioides* from a bog lake and showed that it had the ability to degrade about 60% of the riverine or groundwater humic substances. The same ability was also observed in *Polyporus versicolor*. More interestingly, Rojas-Jimenez et al. [9] showed the ability of *Cladosporium* sp. to simultaneously degrade and synthesize HS. They, similar to Claus and Filip [8], concluded that there is a correlation between the ability of fungi to exhibit lignocellulolytic activity and the degree of their contribution to the degradation/transformation of DOM. However, more studies are needed to overcome limitations of the current results. For example, although some fungi isolated from freshwater ecosystems have shown high enzymatic activity, we still do not know whether these cosmopolitan fungal strains could be considered as representative of endemic fungal communities. Also, in extensive work, Collado et al. [10] listed various recently used measurement methods to assess the degradation of HS by fungi, the variety of which makes comparison problematic to some extent. 

Although oomycetes are among the most common freshwater eukaryotic organisms and found in the same habitats as fungi are, there is a categorical gap of knowledge regarding their underlying ecological contributions. This is mainly due to the fact that they are primarily recognized as very dangerous pathogens to fish, crustaceans, and amphibians [11,12]. However, although a few rare studies have pointed to their implications for the stability of food webs through their interaction with various trophic levels [13], it is not clear whether, enzymatically speaking, they have been equipped with the same properties as fungi. For example, although it has been established that members of the order Saprolegniales are associated with the plant litter in the littoral zone of freshwater ecosystems [14], their precise enzymatic capacity and role in aquatic carbon turnover (degradation of plant litter and HS, in particular) remain to a large extent unknown. In two early studies, however, Saprolegniales taxa exhibited different levels of cellulolytic and chitinolytic activities [15,16]. Solely, in a single study, two strains of the genera *Dictyuchus* and *Aspergillus* (as Saprolegniales and fungal representatives, respectively) were compared for their enzymatic abilities and their contributions to HS degradation and transformation [17]. The genus *Dictyuchus* lacked ligninolytic activity and exhibited a much lower degree of HS degradation and transformation (compared to *Aspergillus*). Yet, for better generalization of the observed metabolic patterns and mechanisms, more strains of Saprolegniales and fungi need to be studied in detail. 

Therefore, in this study, we isolated Saprolegniales and fungal strains from plant litter at the water surface of the littoral zone of 12 temperate lakes in Northeast Germany and determined their taxonomic positions using a set of different marker genes. The cellulo-, lignino-, and chitinolytic activities of all strains were tested to better understand their interactions with plant and animal litter. We further studied the 10 fungal strains with the highest isolation frequency to compare their contributions to the labile carbon pool. Finally, the three strains with the highest enzymatic activity were selected to determine whether they produce and transform HS. This study has important implications for diversity and the ecological role of Saprolegniales and fungi in carbon cycling in the littoral zone of lakes. 

## 2. Materials and Methods

### 2.1. Isolation of Oomycetes and Fungal Strains

Saprotrophic Saprolegniales and fungal strains were isolated from wood and leaves that were collected over several months in 2018 along the shoreline of 12 lakes of Northeast Germany: Breiter Luzin (location A: 53.353333, 13.464913; B: 53.359681, 13.489465), Dagowsee (53.150730, 13.053070), Gerlinsee (53.133643, 12.994251), SW basin of Große Fuchskuhle (53.105700, 12.984735), Großer Stechlinsee (53.152609, 13.022283), Großer Wummsee (53.187389, 12.800203), Kochsee (53.131860, 13.107672), Melzer See (53.526695, 12.701967), Peetschsee (53.167243, 13.075034), Roofensee (53.111463, 13.035540), Tiefwarensee (53.527318, 12.691670), and Wittwesee (53.123667, 12.938442) (Table 1). Isolation of oomycete strains was performed using the baiting method as previously described [18]. For fungal strains, the same plant materials were moist-incubated in Petri dishes and then examined every three days under a binocular (OMAX) to detect their fruiting bodies for several weeks [19]. 

### 2.2. Molecular Characterization of the Strains

DNA extraction followed the protocol of Montero-Pau et al. [20] with minor modifications. Briefly, 100 μL of alkaline lysis buffer (NaOH 25 mmol L^−1^, disodium ETDA 0.2 mmol L^−1^, pH 8.0) was aliquoted into 1.5 mL tubes. A clot of mycelia of fungi and oomycete zoospores growing in the malt extract broth were added to the alkaline lysis buffer and centrifuged for 30 min at 9000 rpm. Samples were incubated at 95 °C for 30 min and then cooled on ice for 5 min. Finally, 100 μL of neutralizing solution (Tris-HCl 40 mmol L^−1^, pH 5.0) was added to the tubes. The samples were vortexed and stored at −20 °C. The small subunit (SSU), ribosomal internal transcribed spacer (ITS), and large subunit (LSU) regions were amplified for Saprolegniales and fungal strains in a Flexible PCR Thermocycler (Analytik Jena, Jena, Thuringia, Germany) using the SSU817/SSU1536, ITS1/ITS4, and LR0R/LR5 primer pairs and the respective PCR conditions [21,22,23]. Only for oomycetes, the *coxI* region was amplified using OomCoxI-Levup/Fm85mod primers following the proposed PCR conditions [24]. All amplification products were kept at −20 °C and sent to a sequencing company (Macrogen, Geumcheon-gu, Seoul, South Korea) for Sanger sequencing. The resulting sequences were edited using BioEdit software [25] and submitted to GenBank (National Center for Biotechnology Information; http://www.ncbi.nlm.nih.gov, accessed on 20 October 2021) (for accession numbers, see Table 1). Molecular classification was performed by comparing strain sequences with those stored in GenBank sequence databases.

### 2.3. Phylogenetic Analysis

The amplified sequences of the strains, together with sequences of the related representative genera, retrieved from GenBank, were used to perform phylogenetic analysis. The sequences were aligned using MAFFT [26] and analyzed with MEGA7 using the maximum likelihood method [27].

### 2.4. Screening for Lignino-, Cellulo-, and Chitinolytic Activity

#### 2.4.1. Ligninolytic Activity

Mycelia from the edge of 7–15-day-old cultures were transferred to six-well culture plates containing the cultivation medium proposed by Rojas-Jimenez et al. [9]. In essence, mPmTG agar medium amended with one of the following substrates was used: 2,20-Azino-bis 3-ethylbenzothiazoline-6-sulfonic acid diammonium salt (ABTS) (0.1% *wt*/*vol*) and Bromocresol Green (BG), Congo Red (CR), Malachite Green (MG), Phenol Red (PhR), PolyR-478 (PR), Remazol Brilliant Blue (RBBR), and Toluidine Blue (TB) (0.02 and 0.005% *wt*/*vol* for each substrate) [17,28]. The capacity of each strain for ligninolytic activity was determined by decolorization (i.e., a halo) of the mentioned substrates in the area around the mycelia or as a color change of the media after three weeks of incubation. A decolorization of the medium by 0–33, 33–66, and 66–100% in all screening treatments was considered as low, medium, and strong, respectively. The area of decolorization was observed and determined by eye. Decolorization of ABTS and other substrates (BG, CR, MG, PhR, PR, and RBBR) are indicators of laccases’ and peroxidases’ production, respectively.

#### 2.4.2. Cellulolytic Activity

The same media used in the evaluation of ligninolytic activity were amended separately with the following carbon sources: 7.5 g carboxymethylcellulose (CMC), 7.5 g Avicel (AVL), and 5 g D-cellobiose (DCB) [28]. After three weeks of incubation, Congo Red (1 mg mL^−1^) was amended to the medium and the plates were incubated at room temperature for 15 min. Subsequently, the medium was rinsed with distilled water and 30 mL of 1 M NaCl added. Degradation of CMC, AVL, and DCB was confirmed by a transparent appearance of the medium (and mycelia) [26] and scored as explained for the ligninolytic activity (see above).

#### 2.4.3. Chitinolytic Activity

Chitinolytic properties of the strains were qualitatively determined using the method proposed by Agrawal and Kotasthane [29]. The final product of crab shell flakes (prepared according to the protocol) was then added to the chitinase detection medium, which consisted of a basal medium comprising (per liter) 0.3 g of MgSO_4_-7H_2_O, 3 g of (NH_4_)_2_SO_4_, 2 g of KH_2_PO_4_, 1 g of citric acid monohydrate, 15 g of agar, 200 uL of Tween−80, 4.5 g of colloidal chitin (CC), and 0.15 g of Bromocresol Purple; the pH was adjusted to 4.7 and the neutralized medium was autoclaved. Degradation of chitin was conducted by measuring the radius of the purple to black area around the mycelia in the center of Petri dishes and scored as explained for the ligninolytic activity (see above).

### 2.5. Physiological Profiling Based on 95 Labile Carbon Sources

Six *Cladosporium* (*Cladosporium* sp. strain FB12, *C. cladosporioides* strains FBP8, FBL81, and FB7, *C. herbarum* strains FB11 and FBPD2) and four *Penicillium* strains (*P. crustosum* strain FBSL1, *P. brevicompactum* strains FBP7, FBP81, and FBP5) were selected for physiological profiling due to their different lignocellulolytic affinities and higher abundance compared to other taxa, using Biolog FF MicroPlates™ (two plates per strain), which contain 95 labile carbon sources (Biolog, Hayward, CA, USA). However, we could not test Saprolegniales as the selected strains failed to produce enough viable zoospores. Furthermore, the mycelia of Saprolegniales are much bigger than that of fungal strains, which could have blocked the wells and thus interrupted the measurements. Twenty mL of the special suspension provided by the manufacturer was poured onto each cultivation plate to ease the detachment of spores. The spore suspension was transferred to a 50 mL Falcon tube using a sterile pipette. To measure the concentration of the spore suspension, spores were counted by a hematocytometer, and a relatively equal concentration for all strains was obtained. A 100 µL suspension of each strain was added to each well of the EcoPlates, which were incubated at 24 °C for 108 h. The activity was measured using a Synergy™ 2 Multi-Mode Microplate Reader (BioTek, Winooski, VT, USA) set for absorbance at 590 nm every 12 h.

Two parameters are used to describe the utilization of carbon sources by strains: average metabolic response (AMR) and strain metabolic diversity (SMD). AMR is the average respiration of the C-sources and is calculated as the average of the mean difference between the O.D. of the C-source-containing wells and the control well. SMD is analogous to the functional richness of strains and is calculated by summing the number of positive responses (purple-colored wells) observed following incubation. In fact, AMR and SMD show how fast and diverse any given strain utilizes the 95 different C-sources.

### 2.6. DOM Utilization and HS Degradation and Production

The experimental setup was adopted from Rojas-Jimenez et al. [9] and Masigol et al. [17] and further improved. In particular, the number of treatments was increased to test the main and interactive effects of maltose and humic acid as labile and refractory carbon sources, respectively. Three strains were selected for the experiment for two main reasons. Firstly, *Phlebia* sp. ASW4, *Mortierella* sp. AFL4-2, and *Saprolegnia* sp. MSK3-3 showed the highest levels of enzymatic activity. Also, the ASW4, AFL4-2, and MSK3-3 strains are representative of different taxa: Basidiomycota, Mucoromycota, and Oomycota (Saprolegniales), respectively.

The following combinations were used to carry out the experiment: Treatment (T) 1 (MA (stands for maltose)): Test medium [10] and 1 g maltose as sole carbon source; T2 (MA + Fe (maltose and iron)): Test medium, 1 g maltose, and 10 mg FeSO_4_, where briefly, FeSO_4_ was added to test whether Fenton reaction is involved in carbon utilization; T3 (HS (humic substances)): Test medium and 50 mg humic substances from Lake Große Fuchskuhle; T4 (HS + Fe): Test medium, 50 mg humic substances from Lake Große Fuchskuhle, and 10 mg FeSO_4_; T5 (Ma + HS (maltose and humic substances)): Test medium, 1 g maltose, and 50 mg humic substances from Lake Große Fuchskuhle; T6 (MA + HS + Fe (maltose, humic substances, and iron)): Test medium, 1 g maltose, 50 mg humic substances from Lake Große Fuchskuhle, and 10 mg FeSO_4_. The three strains with the highest enzymatic activity were chosen for the experiment. The *Phlebia* sp. strain ASW4 (Basidiomycota) and *Mortierella* sp. strain AFL4-2 (Mucoromycota), as representatives of fungi, and the *Saprolegnia* sp. strain MSK3-3, as a representative of Saprolegniales, were separately inoculated in the abovementioned media and incubated for 21 days at room temperature with constant shaking (100 rpm). Four replicates with respective controls were considered for each treatment.

After the incubation period, a total organic carbon analyzer (TOC-VCPH, Shimadzu) was used to measure the dissolved organic carbon (DOC) content of a 1:100 dilution of each treatment, and the method of so-called liquid chromatography-organic carbon detection-organic nitrogen detection (LC-OCD-OND) [30] was deployed to separate and quantify major classes of natural organic matter (NOM) [31]. In addition to the total DOC, three DOC-related parameters were considered, i.e., low molecular weight substances (LMWS), HS production, and the spectral absorption coefficient (SAC 254 nm). LMWS confirms that the utilized DOC stems from the carbon source amended in the treatments. HS production indicates whether any of the tested strains could have degraded the provided carbon source(s) and subsequently transformed it to HS. Also, SAC 254 nm is an integrative parameter measuring the DOM that absorbs UV light at a wavelength of 254 nm. A reduction or an increase in SAC 254 nm causes an alteration in the aromaticity of humic substances, which suggests microbial transformation of HS.

### 2.7. Statistical Analysis

In regard to the Biolog assays, a threshold optical density (OD) was established at OD_590_ > 0.25 according to the manufacturer’s instructions. For each substrate replicate, we subtracted the average blank measurement and then estimated the substrate utilization, which we calculated by integrating the area under the curve (AUC), where the OD_590_ exceeded 0.25 [32]. For each substrate, we averaged the AUC across the three replicates. The average metabolic response (AMR) and the strain metabolic diversity (SMD) of each strain were calculated by averaging the AUC for all positive substrates and summing the number of positive responses (OD_590_ > 0.25) (purple-colored wells) observed following incubation, respectively. We tested for significant differences in the metabolic capabilities between strains across all substrates using a permutational multivariate analysis of variance (PERMANOVA). Substrate-specific metabolic differences between strains were assessed using a two-way ANOVA, with subsequent pairwise assessments, accounting for multiple comparisons, as assessed using Tukey’s test.

## 3. Results

### 3.1. Isolation and Identification

**Figure 1:**
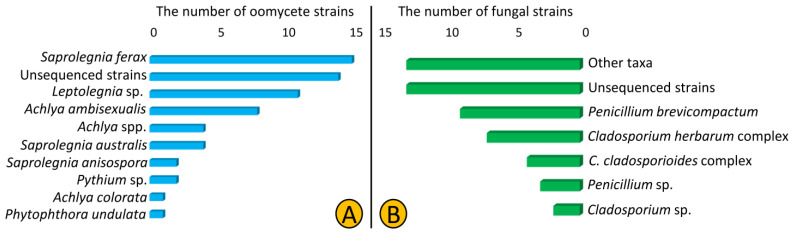
Composition of oomycete (Saprolegniales and Peronosporales) (**A**) and fungal (**B**) taxa isolated from 12 lakes of Northeast Germany in 2018.

In total, we isolated 62 oomycetes (Saprolegniales and Peronosporales) and 51 fungal strains from 12 lakes of Northeast Germany using culture-dependent methods. The SSU and *CoxI* primers used in this study were not efficient as only 23 and 25% of isolated oomycete and fungal strains were amplified, respectively. According to the blast results of the obtained ITS sequences, we predominantly isolated strains from Saprolegniales (59 out of 62) that belonged to *Saprolegnia*, *Achlya*, and *Leptolegnia* (34, 21, and 18%, respectively), but also isolated two *Pythium* strains and one *Phytophthora* strain from the order Peronosporales. Additionally, 14 strains (22%) were named unsequenced Saprolegniales as we failed to amplify the ITS region (Table 1, Figure 1A). Regarding fungal strains, the *Cladosporium* and *Penicillium* strains were the most frequently isolated (24 and 25% each). We assigned most of strains (26%) to the genus and higher levels as the blasting results from the sequenced regions were ambiguous (Figure 1B).

**Figure 2:**
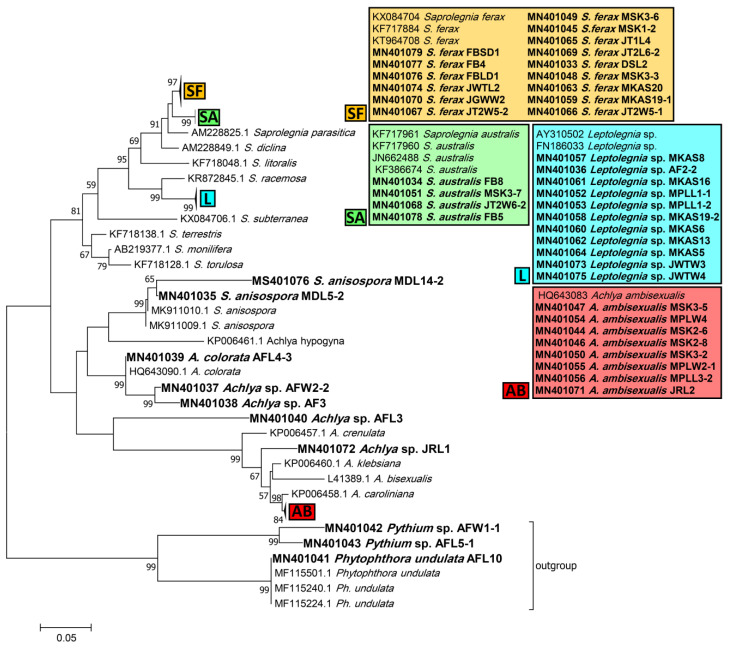
Phylogenetic tree of obtained Saprolegniales and Peronosporales isolates inferred from the analysis of the aligned ITS1-5.8S-ITS2 sequences (666 bp) using the maximum likelihood method from strains in this study and validated sequences from GenBank. The numbers next to the branches show bootstraps values ≥ 50%. *Ph. undulata* (MF115501, MF115240, and MF115224) was considered an outgroup.

Phylogenetic trees inferred from ITS sequences were used to assign the Saprolegniales and Peronosporales strains in this study to their corresponding taxa. According to the ITS tree, *Saprolegnia* strains were closely affiliated to *Saprolegnia ferax*, *S. australis*, and *S. anisospora* (15, 4, and 2 strains, respectively). *Achlya* strains were related to *Achlya ambisexualis* and *A. colorata* (8 strains and 1 strain, respectively), while four remained unassigned to a species, i.e., *Achlya* spp. Also, none of the *Leptolegnia* and *Pythium* strains (11 and 2, respectively) could be assigned to any species. Finally, one *Phytophthora* strain was assigned to *Phytophthora undulata* (Figure 2).

**Figure 3:**
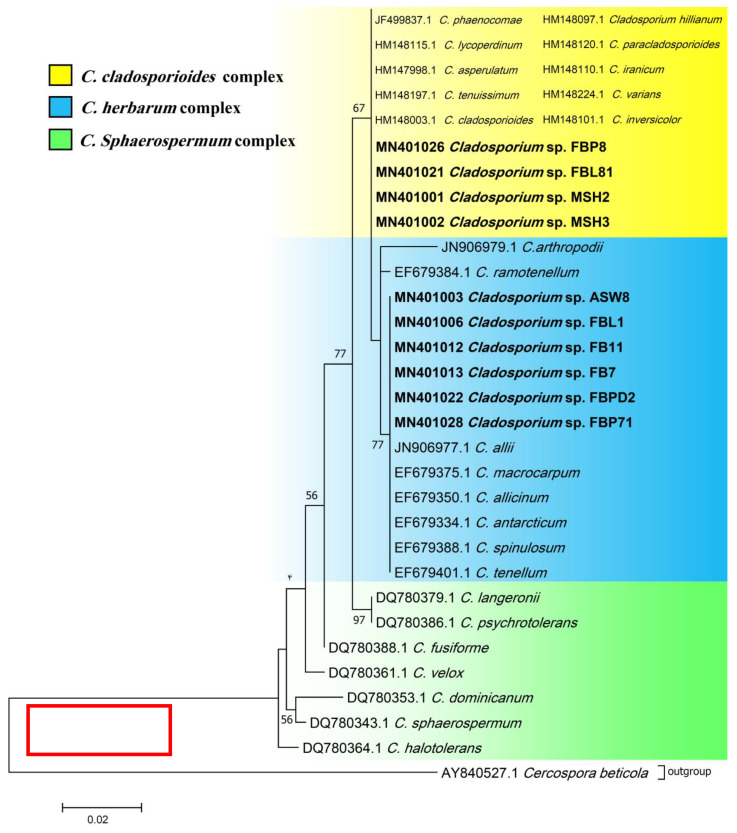
ITS phylogenetic trees of the genus *Cladosporium* and related genera. The analyses were performed based on the alignments of ITS1-5.8S-ITS2 (453 bp) using the maximum likelihood method from isolates in this study (bold) and validly published sequences from GenBank. The numbers next to the branches show bootstraps values ≥ 50%. *Cercospora beticola* was considered an outgroup.

**Figure 4:**
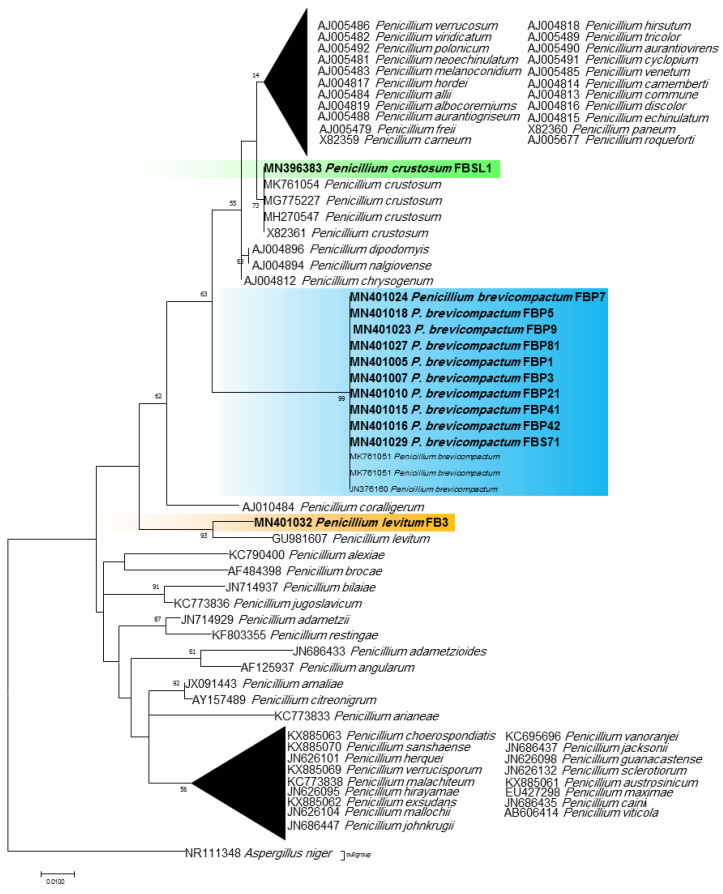
ITS phylogenetic trees of the genus *Penicillium* and related genera. The analyses were performed based on the alignments of ITS1-5.8S-ITS2 (502 bp) using the maximum likelihood method from isolates in this study (bold) and validly published sequences from GenBank. The numbers next to the branches show bootstraps values ≥ 50%. *Aspergillus niger* was considered an outgroup.

Phylogenetic ITS and LSU trees were considered for *Cladosporium* and *Penicillium* due to their prominent occurrence in the fungal strains. *Cladosporium* strains FBP8, FBL81, MSH2, and MSH3 were grouped in the *C. cladosporioides* complex, and strains ASW8, FBL1, FB11, FB7, FBPD2, and FBP71 in the *C. herbarum* complex according to ITS sequences (Figure 3). The results from the LSU sequences were not clear enough as strains were placed in a clade including several *Cladosporium* species (the tree is not shown). Additionally, all *Penicillium* strains clustered with *P. brevicompactum*, except for FBSL1 and FB3, based on both ITS and LSU sequences (LSU tree is not shown) (Figure 4).

### 3.2. Ligninolytic, Cellulolytic, and Chitinolytic Activity

**Figure 5:**
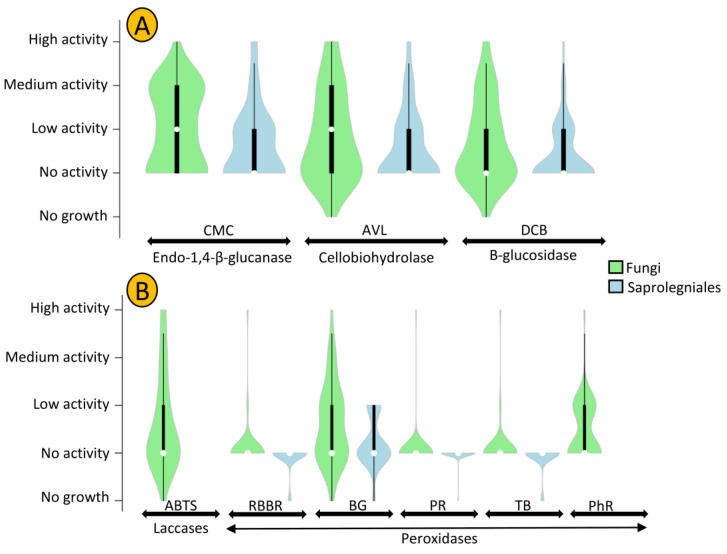
The levels of cellulolytic (**A**) and ligninolytic (**B**) activity of oomycete and fungal strains isolated in this study. CMC (carboxymethylcellulose), AVL (Avicel), and DCB (D-cellobiose) are substrates amended in cultures to detect the presence of enzymes involved in cellulolytic activity. ABTS, RBBR, BG, PR, TB, and PhR are substrates amended in cultures to assess the presence of enzymes involved in ligninolytic activity. White circles show the medians; box limits indicate the 25th and 75th percentiles as determined by R software; whiskers extend 1.5 times the interquartile range from the 25th and 75th percentiles; polygons represent density estimates of data and extend to extreme values.

**Figure 6:**
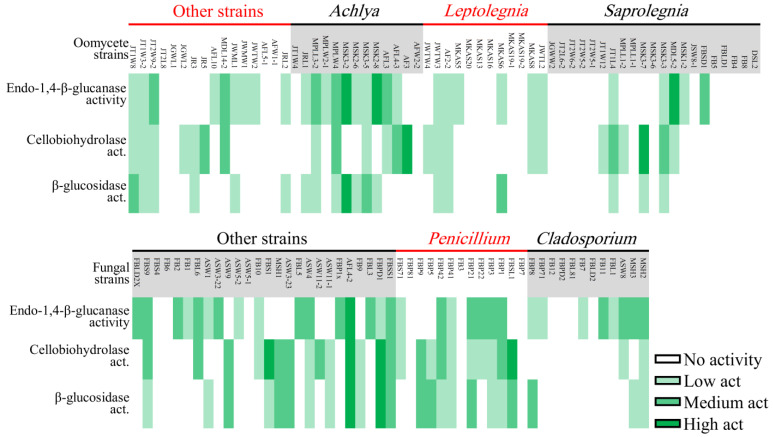
Heatmap showing cellulolytic activity of oomycete (**upper**) and fungal (**lower**) strains isolated in this study.

The cellulolytic activity of strains was assessed using CMC (carboxymethylcellulose), AVL (Avicel), and DCB (D-cellobiose), which showed the presence of endo-1,4-β-glucanase, cellobiohydrolase, and β-glucosidase enzymes, respectively. In general, fungal strains revealed a higher level of cellulolytic activity compared to oomycete strains (Figure 5A). *Mortierella* sp. AFL-2 and *Achlya* sp. MSK3-2 showed the highest levels of cellulolytic activity among fungal and oomycete strains, respectively. In total, 39% of all oomycetestrains exhibited no cellulolytic activity, while this number was only 20% in fungal strains (Figure 6).

*Penicillium* and *Achlya*, which were the most frequently isolated genera, were more likely to exhibit cellulolytic activity, with 71 and 77% of their strains positive for at least two of the tested enzymes. We detected activity of at least two enzymes for 42% of *Leptolegnia* and 33% of *Cladosporium* strains (Figure 6). Oomycete and fungal strains differed in their capacity to degrade lignin, as evaluated through the detection of laccase and peroxidase activity (Figure 5B). Laccase activity was observed in 37% of our fungal but in none of the oomycete strains. Additionally, a higher capacity was observed in fungal strains for the production of peroxidases. Nearly 47 and 41% of fungal strains could decolorize Bromocresol Green (BG) and Phenol Red (PhR), compared to 27 and 0% of oomycete strains. When considering both cellulo- and ligninolytic activity, only 9% of isolated fungi and 24% of oomycete strains exhibited no observable enzymatic activity. *Phlebia* sp. ASW4 was the most enzymatically active strain as it produced the full array of both tested cellulo- and ligninolytic enzymes.

Nearly 66 and 76% of oomycete and fungal strains exhibited chitinolytic activity. In oomycete, 73% of *Leptolegnia* sp. strains showed moderate to high chitinolytic activity followed by *Achlya* (71%) and *Saprolegnia* (41%). Among fungi, chitinolytic activity was detected in 92 and 83% of *Penicillium* and *Cladosporium* strains. Therefore, chitinolytic activity was higher than cellulolytic and ligninolytic activity in both fungi and oomycete strains. 

### 3.3. Physiological Profiling of Strains Based on Utilization of 95 Labile Carbon Sources

**Figure 7:**
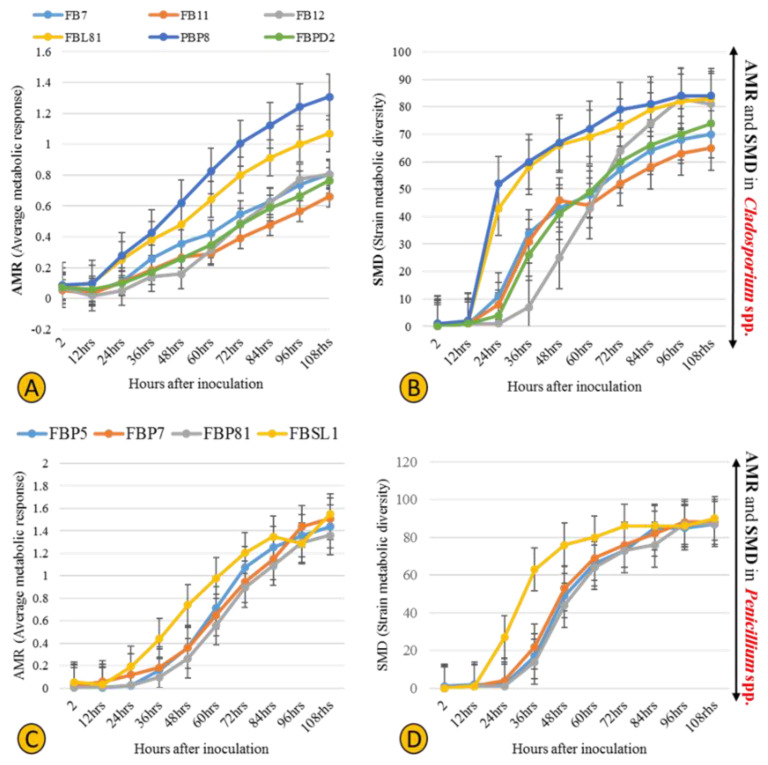
Physiological profiles of six *Cladosporium* and four *Penicillium* strains isolated in this study, with the AMR (average metabolic response) (**A**,**C**) and SMD (strain metabolic diversity) (**B**,**D**).

**Figure 8:**
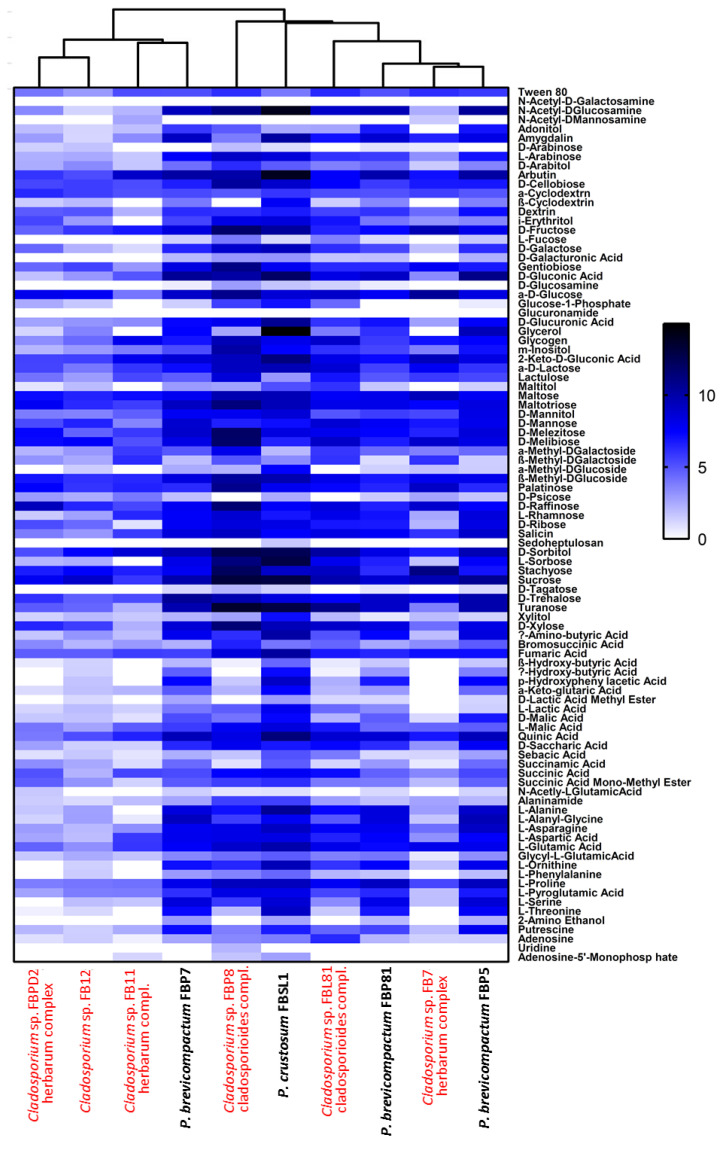
Comparative heatmap of *Cladosporium* and *Penicillium* strains grown on Biolog FF MicroPlates™ after 108 consecutive hours based on the utilization of 95 different carbon sources with two replicates each. The white to black color gradient indicates poor to high growth on the respective substrates (color scale = specific AUC value).

Six *Cladosporium* and four *Penicillium* strains were selected for their different lignocellulolytic affinities and higher abundance compared to other genera. The final measured AMR (average metabolic response) and SMD (strain metabolic diversity) parameters were not analogous in *Cladosporium* strains, ranging from 0.66 to 1.3 and 65 to 84, respectively, (Figure 7A,B) with FBP8 and FB11 as the most and least active strains. *Penicillium* strains exhibited a similar trend; the AMR and SMD parameters’ ranges of change were 1.35–1.54 and 87–90, respectively (Figure 7C,D). The only outlier was FBSL1, which showed a higher AMR and SMD during the experiment, but ended up in a position similar to other *Penicillium* strains. In general, the average respiration of the C-sources (calculated by AMR) and the number of substrates utilized (calculated by AMR) by *Cladosporium* strains were higher than for *Penicillium* strains. The heatmap in Figure 8 shows how each strain utilized 95 C-sources. According to the diversity and quality of substrates’ utilization, strains were classified in two groups: *Cladosporium* sp. strains FBPD2, FB12, FB11, and *Penicillium* sp. strain FBP7 in one group with a lower utilization rate, and the rest (*Penicillium* strains FBP81, FBSL1, FBP5, FBP81, and *Cladosporium* strains FBP8, FBL81, and FB7) in the other group with a higher utilization rate. None of the strains could utilize N-acetyl-D-galactosamine and glucuronamide. Also, sedoheptulosan and uridine were only utilized by one strain. In contrast, sucrose, D-melezitose, D-melibiose, maltose, and maltotriose were among the most favorable C-sources.

### 3.4. DOM Utilization and HS Degradation/Production

**Figure 9:**
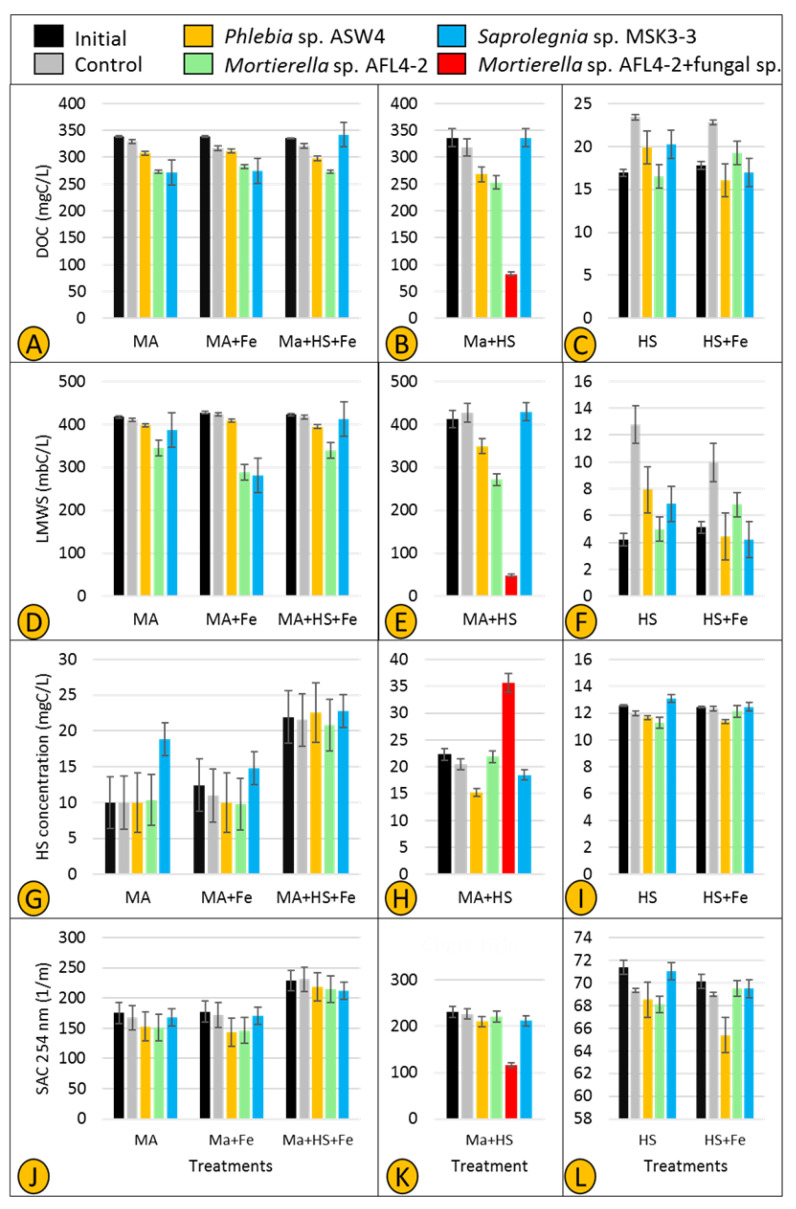
Concentration of dissolved organic carbon (DOC) (**A**–**C**), low molecule weight substances (LMWS) (**D**–**F**), and the levels of HS (including building blocks) (**G**–**I**) and SAC 254 nm (**J**–**L**) in six treatments (see material and methods section for more detail) inoculated separately by two fungal strains (*Phlebia* sp. ASW4 and *Mortierella* sp. AFL4-2) and one Saprolegniales strain (*Saprolegnia* sp. MSK3-3), along with *Mortierella* sp. AFL4-2 + fungal sp., after 21 days.

Two parameters—DOC and LMWS—were used to investigate whether fungal and Saprolegniales isolates show specific utilization of carbon compounds. All three selected strains (*Phlebia* sp. ASW4, *Mortierella* sp. AFL4-2, and *Saprolegnia* sp. MSK3-3) slightly utilized maltose as the sole carbon source in treatments MA and MA + Fe (Figure 9A). However, when both maltose and HS were present (in treatment MA + HS + Fe), utilization of maltose by *Saprolegnia* sp. MSK3-3 was completely inhibited. The rate of maltose utilization remained unchanged for *Phlebia* sp. ASW4 and *Mortierella* sp. AFL4-2 (methodological consideration (MC): a small loss of DOC was observed in treatments MA, MA + Fe, and MA + HS + Fe controls, which was possibly the result of maltose binding to the agar inoculum).

In treatment MA + HS (Figure 9B), the utilization rate was intensified so that the combination of *Mortierella* sp. AFL4-2 + fungal sp. showed the lowest MA value followed by *Mortierella* sp. ALF4-2 and *Phlebia* sp. ASW4, respectively. Similar to treatment MA + HS + Fe, *Saprolegnia* sp. MSK3-3 showed no maltose utilization (Figure 9B). In accordance with the DOC concentration, LMWS was found to be decreased in treatments MA, MA + Fe, and MA + HS + Fe inoculated with *Phlebia* sp. ASW4 and *Mortierella* sp. AFL4-2. Also, the LMWS concentrations decreased only in treatment MA + Fe inoculated with *Saprolegnia* MSK3-3 (Figure 9D). The intensification of DOC utilization was confirmed by a very sharp decrease in treatment MA + HS inoculated with *Mortierella* sp. + fungal sp. (Figure 9E) (MC: the performance of strains was generally inconsistent in treatments HS and HS + Fe. In particular, controls had higher DOC concentrations in the treatments HS and HS + Fe (Figure 9C,F), a possible result of the agar plugs leaching DOC into the medium).

HS production and SAC 254 nm were also used to assess possible production/transformation of HS. An increase in the level of HS was observed in treatment MA inoculated only by *Saprolegnia* sp. MSK3-3 (Figure 9G), while the SAC 254 nm value did not substantially change (Figure 9J). In treatments MA + Fe and MA + HS + Fe, neither the level of HS nor SAC 254 nm consistently changed. However, a very clear increase and decrease in the levels of HS and SAC 254 nm, respectively, were recorded in treatment MA + HS inoculated with *Mortierella* sp. AFL4-2 + fungal sp. (Figure 9H,K). Also, *Phlebia* sp. ASW4 could decrease HS (Figure 9H), but SAC 254 nm remained nearly unchanged (Figure 9K) (MC: an increase was observed in the value of HS in all initials and controls of the MA + HS + Fe treatments, which might be the result of an interaction between maltose and HS (Figure 9G)).

## 4. Discussion

In this study, we isolated 62 and 51 oomycete and fungal strains isolated from 12 temperate lakes in Northeast Germany and studied their phylogeny, cellulo- , chitin-, and ligninolytic activities, ability to utilize different labile carbon sources, and involvement in humic substances’ production and transformation. Most fungal strains belonged to the *Cladosporium* and *Penicillium* genera, while *Saprolegnia*, *Achlya*, and *Leptolegnia* were the most frequently isolated Saprolegniales. Whilst most fungal and oomycete strains exhibited cellulolytic activity, only *Cladosporium* and *Penicillium* strains exhibited ligninolytic activities that would account for HS turnover in aquatic ecosystems. *Cladosporium* and *Penicillium* broadly exhibited a high capacity to utilize a variety of labile and recalcitrant carbon sources. Finally, a combination of *Mortierella* sp. + fungal sp. in treatment MA + HS resulted in the highest maltose utilization, above that of any other strains, and also contributed most to the production of HS. The important ecological role of both fungi and Saprolegniales in freshwater carbon cycling was confirmed and discussed in this study. Although cosmopolitan genera (*Cladosporium* and *Penicillium* strains isolated in our case) have been previously isolated from different freshwater ecosystems [9,33,34], they cannot be inherently called aquatic fungi for two main reasons. Firstly, the origin of such strains is hard to track as they may randomly enter the littoral zone of freshwater ecosystems when associated with plant litter input. Secondly, their dissemination agents—spores, in particular—are not adapted to life in the water [35]. Therefore, even though they showed high cellulo- and to a lesser extent ligninolytic activities in our study, the degree and magnitude of their contribution to plant litter degradation remain to a large extent unknown. Therefore, it is suggested that we should conduct extensive environmental DNA sampling in different environmental niches and seasons, to determine whether cosmopolitan genera substantially contribute to fungal communities associated with plant litter in the littoral zone of inland waters. In contrast to the tested fungal strains, our isolated *Achlya, Saprolegnia*, and *Leptolegnia* strains are exclusively aquatic and very rarely found in terrestrial habitats. The presence of these three Saprolegniales genera in a variety of freshwater ecosystems across the globe has been known for many years [36,37]. However, the accurate taxonomic positioning and species diversity of these strains are often biased. In our study, we thus tried to simultaneously amplify ITS, LSU, and *cox1* regions using the ITS1/ITS4, LR0R/LR5, and OomCoxI-Levup/Fm85mod primer pairs, respectively, to uncover yet hidden diversity via a multi-gene phylogenetic approach. Unfortunately, with the exception of the ITS region, amplification of the other two marker regions was not successful. We believe this might be the case because most primers have been primarily designed and optimized for *Peronosporales* and *Pythiales*. According to the ITS phylogenetic tree, some *Achlya* and *Saprolegnia* sp. and all *Leptolegnia* strains could not be assigned to any known species and thus might be candidates for new species. Nevertheless, it cannot be tested reliably with the ITS marker sequence alone or when sequenced full genomes are not available. Therefore, we propose that a more Saprolegniales-friendly approach is required to resolve species boundaries efficiently. In addition, approaches such as environmental DNA sampling are of great importance to better understanding Saprolegniales’ diversity and occurrence in defined environmental niches. Currently, colonization and interaction of saprophytic and pathogenic strains on plant litter in the littoral zone of lakes have been poorly studied, but this can be resolved by improved environmental DNA sampling approaches. 

In most cases, our fungal strains could produce at least one enzyme involved in the degradation of cellulose-containing fractions of plant litter. However, fingerprints of cellulolytic activities of phylogenetically identical strains (*Cladosporium* and *Penicillium*, in particular) varied with the degree of litter degradation. Interestingly, although higher lignin and cellulose degradation has mainly been affiliated with brown rot fungi (Basidiomycota) [38], our *Mortierella* sp. AFL4-2 strain—with the highest cellulolytic activity of all of our tested strains—belongs to Mucormycota. Although *Mortierella* has been reported a few times as a potential cellulose degrader [39,40], it is often ignored in culture-based studies where strains belonging to Asco- and Basidiomycota have been isolated much more frequently. Nevertheless, it seems that communal efforts of entire fungal communities (including strains from different phyla) associated with plant litter substantially accelerate the decomposition of cellulose-based materials [41]. As mentioned earlier, an improved metabarcoding approach is needed to estimate the contribution of each taxon to the degradation of cellulose-containing plant litter in freshwater ecosystems. 

Our oomycete strains showed some cellulolytic activity, but less pronounced when compared to fungal strains. Since both oomycete and fungal strains frequently colonize plant litter, one could argue that both compete for cellulose-based materials. Previously, Unestam [15] and Thompson and Dix [42] pointed to a lack of or moderate cellulolytic activity in some Saprolegniales taxa. More recently, Masigol et al. [17,43] showed a wide range of cellulolytic activity occurs in taxa belonging to the Saprolegniales genera such as *Achlya*, *Dictyuchus*, and *Saprolegnia*. Similar to our fungal strains, a large variation regarding cellulolytic capabilities was observed even in phylogenetically identical strains. Thus, profiling the cellulolytic activity of Saprolegniales taxa might help to better define the taxonomy of strains when morphometric and molecular data fail to do so. 

Our oomycete and fungal strains greatly differed in terms of lignin degradation. The production of laccases and peroxidases (involved in depolymerizing lignin) was observed in several fungal strains, but less frequently than with cellulolytic enzymes. It should be noted that two of the most active lignin degraders among our fungal strains (*Heterobasidion* sp. FBL3 and *Phlebia* sp. ASW4 (*Basidiomycota*)) are among common representatives of terrestrial and freshwater white rot fungi [44,45]. For example, *Phlebia tremellosa* has been reported as an effective degrader of the water reed *Phragmites communis*, CNP herbicide, industrial dyes, the organochlorine pesticide dieldrin, and DDT from several anthropogenically contaminated freshwater ecosystems [46].

In contrast, the lack of any laccase and very low production of peroxidases by our tested Saprolegniales strains confirms previous results from freshwater and brackish systems [17,43]. Laccases significantly contribute to the pathogenicity and defense mechanisms of phytopathogenic fungi [47]. Also, laccase genes have been identified in four terrestrial phytopathogenic oomycetes species [48,49,50,51], confirming the importance of laccases for their pathogenicity. This enzyme, for example, is responsible for the pathogenicity of *Plasmopara viticola* in grapevine [52]. This notion also confirms recent in-depth phylogenetic studies indicating that laccases and many other polymer-degrading enzymes have been acquired by oomycetes pathogens via horizontal or endosymbiotic gene transfer [53]. The absence of these enzymes in aquatic Saprolegniales suggests their reduced potential to degrade lignin-based materials in their natural habitats. The retrieved ligninolytic inefficacy of aquatic Saprolegniales points to important ecological implications and the ecological and evolutionary diversification of oomycetes in the aquatic vs. terrestrial realms [54].

Finally, although extensive sampling of different types of plant litter from different lakes was conducted, Saprolegniales strains were not highly diverse. Thus, it is not reasonable to make generalizations from a handful of genera. In addition to *Saprolegnia* and *Achlya*, other genera such as *Dictyuchus*, *Brevilegnia*, etc., should be isolated and studied in detail to determine whether there are general trends in metabolic capabilities among all members of Saprolegniales [55].

The high physiological variability among *Cladosporium* and *Penicillium* strains emphasizes the interaction of fungal strains in utilizing labile organic matter. Hypothetically, degradation of plant litter by different fungi gradually leads to a substantial release of labile organic matter, which is subsequently taken up by additional fungal strains. These easy-to-digest carbon sources will thus boost various fungal strains to also degrade recalcitrant organic matter until the amount of extracellular HS increases. When providing maltose as a sole carbon source, it was used by the Saprolegniales strain *Saprolegnia* MSK3-3 more efficiently than the two tested fungal strains (Figure 9A). This is in accordance with the study of Masigol et al. [17], which shows that *Dictyuchus* strains (Saprolegniales) take up labile organic matter more efficiently than several fungal strains. Consequently, we believe that both fungal and Saprolegniales strains compete over labile organic matter as they do for the more hard-to-degrade cellulose-containing organic matter.

Fungi are believed to play a key role in the process of humification in soil, and more recently, freshwater ecosystems. Rojas-Jimenez et al. [8] and Masigol et al. [16] proved this notion for *Cladosporium* and *Aspergillus* sp. strains isolated from two different freshwater ecosystems. Interestingly, one of the major findings of this study was obtained accidentally, i.e., we detected a source of fungal contamination (*Aspergillus* based on colony morphology) in two replicates of treatment MA + HS inoculated with *Mortierella* sp. AFL4-2. We kept these contaminated replicates, named them *Mortierella* sp. AFL4-2 + fungal sp. (Figure 9B,E,H,K), and measured the HS dynamics in comparison to those solely of *Mortierella* sp. AFL4-2. It turned out that *Mortierella* sp. AFL4-2 + fungal sp. represents the most active treatment for organic matter turnover and transformation (as indicated by lower levels of DOC, LMWS, and SAC 254 nm and high levels of HS compared to *Mortierella* sp. AFL4-2 alone and the other individually tested strains). Consequently, the combination of two fungal strains resulted in efficient utilization of maltose and HS, and HS transformation, as indicated by the increased HS concentration and sharp decrease in SAC 254 nm, i.e., changes in HS aromaticity. However, it is not clear whether the contaminant (the fungal sp.) just supported *Mortierella* AFL4-2 to utilize maltose and produce HS more efficiently or acted as another utilizer and/or producer. This needs to be tested as, unfortunately, we did not have any controls for the contaminant. Such an interaction between different fungi might suggest that the capacity of fungal and Saprolegniales strains—for the utilization and transformation of DOM, particularly HS, in freshwater ecosystems—increases in more complex microbial communities. Together with the high cellulolytic activity of *Mortierella* sp. AFL4-2 (Figure 6), the presence of an additional fungus may thus compensate for the lack of ligninolytic activity of the *Mortierella* strain, favoring a high accumulation of humic substances in parallel with high maltose utilization. Such a synergistic behavior has been previously shown for fungal and bacterial strains [56]. These results indicate that single-strain experiments cannot be 1:1 translated to more complex microbial communities *in situ.* To evaluate the “true” ecological role of fungal and oomycete communities, microbial interactions and metabolic linkages need to be taken into account in future studies.

## 5. Conclusions

In this study, we contributed to the knowledge of fungal and oomycete communities associated with floating plant litter in the littoral zone of freshwater ecosystems. Our results suggest that although they occupy the same habitat, Saprolegniales contrasts with fungi by showing no ligninolytic activity. The lack of ability in genera such as *Saprolegnia*, *Achlya*, and *Leptolegnia* to degrade lignin might be explained by their habitat and lifestyle. Regarding the habitat, it can be argued that fungi initiate the lignin degradation process and make less recalcitrant polymers (cellulose, hemicellulose, etc.) more accessible for saprophytic Saprolegniales. Genera such as *Achlya*, with a saprophytic lifestyle and high cellulolytic activity, can be candidates to continue plant litter degradation alongside fungi. Saprolegniales with more flexibility toward parasitism/saprophytism, such as *Saprolegnia*, might be involved in the degradation to a lesser extent. Additionally, we found that Saprolegniales might not interact with humic substances such as recalcitrant organic matter as fungi do. The importance of such interactions lies in the fact that humic substances are involved in many biogeographical processes involved in aquatic carbon sequestration. One reason might be the toxicity of humic substances against Saprolegniales. In our study, a combination of two fungal strains not only produced humic substances, but also changed their aromaticity nature. Such synergistic efforts between different genera of fungi and Saprolegniales must be studied to determine their accurate involvement in the degradation process as well as humic substances’ degradation/production/transformation. 

## Figures and Tables

**Table 1:** Fungal and oomycete strains isolated from 12 temperate lakes in Northeast Germany.

Strain	Taxonomy	Sampling Site	GenBank Accession Number			
			SSU	ITS	LSU	*coxI*
**Oomycetes**						
DSL2	*Saprolegnia ferax*	Großer Stechlinsee	-	MN401033	-	-
FB8	*Saprolegnia australis*	Großer Stechlinsee	-	MN401034	-	-
FB4	*Saprolegnia ferax*	Großer Stechlinsee	-	MN401077	-	-
FBLD1	*Saprolegnia ferax*	Großer Stechlinsee	-	MN401076	-	-
FB5	*Saprolegnia australis*	Großer Stechlinsee	-	MN401078	-	-
FBSD1	*Saprolegnia ferax*	Großer Stechlinsee	-	MN401079	-	-
JSW8-1	Unknown Saprolegniales	Großer Stechlinsee	-	-	-	-
MDL14-2	*Saprolegnia anisospora*	Dagowsee	-	MS401076	-	MN398165
MDL5-2	*Saprolegnia anisospora*	Dagowsee	-	MN401035	-	MN398166
AF2-2	*Leptolegnia* sp.	Große Fuchskuhle	-	MN401036	-	MN398167
AFW2-2	*Achlya* sp.	Große Fuchskuhle	-	MN401037	-	MN398168
AF3	*Achlya* sp.	Große Fuchskuhle	-	MN401038	-	-
AFL4-3	*Achlya colorata*	Große Fuchskuhle	-	MN401039	-	-
AFL3	*Achlya* sp.	Große Fuchskuhle	-	MN401040	-	-
AFL10	*Phytophthora undulata*	Große Fuchskuhle	-	MN401041	-	MN398169
AFW1-1	*Pythium* sp.	Große Fuchskuhle	-	MN401042	-	MN398170
AFL5-1	*Pythium* sp.	Große Fuchskuhle	-	MN401043	-	MN398171
MSK2-6	*Achlya ambisexualis*	Breiter Luzin A	-	MN401044	-	-
MSK1-2	*Saprolegnia ferax*	Breiter Luzin A	-	MN401045	-	-
MSK2-8	*Achlya ambisexualis*	Breiter Luzin A	-	MN401046	-	MN398172
MSK3-5	*Achlya ambisexualis*	Breiter Luzin B	-	MN401047	-	-
MSK3-3	*Saprolegnia ferax*	Breiter Luzin B	-	MN401048	-	MN398173
MSK3-6	*Saprolegnia ferax*	Breiter Luzin B	-	MN401049	-	-
MSK3-2	*Achlya ambisexualis*	Breiter Luzin B	-	MN401050	-	-
MSK3-7	*Saprolegnia australis*	Breiter Luzin B	-	MN401051	-	-
MPLL1-1	*Leptolegnia* sp.	Peetschsee	-	MN401052	-	-
MPLL1-2	*Leptolegnia* sp.	Peetschsee	-	MN401053	-	-
MPLW4	*Achlya ambisexualis*	Peetschsee	-	MN401054	-	-
MPLW2-1	*Achlya ambisexualis*	Peetschsee	-	MN401055	-	-
MPLL3-2	*Achlya ambisexualis*	Peetschsee	-	MN401056	-	-
MKAS8	*Leptolegnia* sp.	Kochsee	-	MN401057	-	-
MKAS19-2	*Leptolegnia* sp.	Kochsee	-	MN401058	-	-
MKAS19-1	*Saprolegnia ferax*	Kochsee	-	MN401059	-	-
MKAS6	*Leptolegnia* sp.	Kochsee	-	MN401060	-	MN398174
MKAS16	*Leptolegnia* sp.	Kochsee	-	MN401061	-	-
MKAS13	*Leptolegnia* sp.	Kochsee	-	MN401062	-	-
MKAS20	*Saprolegnia ferax*	Kochsee	-	MN401063	-	MN398175
MKAS5	*Leptolegnia* sp.	Kochsee	-	MN401064	-	-
JT1W3-2	Unknown Saprolegniales	Tiefwarensee	-	-	-	-
JT1W8	Unknown Saprolegniales	Tiefwarensee	-	-	-	-
JT1L4	*Saprolegnia ferax*	Tiefwarensee	-	MN401065	-	-
JT1W12	Unknown Saprolegniales	Tiefwarensee	-	-	-	MN398163
JT1W4	Unknown Saprolegniales	Tiefwarensee	-	-	-	MN398164
JT2L8	Unknown Saprolegniales	Melzer See	-	-	-	-
JT2W9-2	Unknown Saprolegniales	Melzer See	-	-	-	-
JT2W5-1	*Saprolegnia ferax*	Melzer See	-	MN401066	-	-
JT2W5-2	*Saprolegnia ferax*	Melzer See	-	MN401067	-	-
JT2W6-2	*Saprolegnia australis*	Melzer See	-	MN401068	-	-
JT2L6-2	*Saprolegnia ferax*	Melzer See	-	MN401069	-	MN398176
JGWL2	Unknown Saprolegniales	Gerlinsee	-	-	-	-
JGWL1	Unknown Saprolegniales	Gerlinsee	-	-	-	-
JGWW2	*Saprolegnia ferax*	Gerlinsee	-	MN401070	MN401080	-
JRL2	*Achlya ambisexualis*	Roofensee	-	MN401071	MN401081	-
JR5	Unknown Saprolegniales	Roofensee	-	-	-	-
JR3	Unknown Saprolegniales	Roofensee	-	-	-	-
JRL1	*Achlya* sp.	Roofensee	-	MN401072	-	-
JWTW3	*Leptolegnia* sp.	Wittwesee	-	MN401073	MN401082	-
JWTL2	*Saprolegnia ferax*	Wittwesee	-	MN401074	MN401083	-
JWTW4	*Leptolegnia* sp.	Wittwesee	-	MN401075	MN401084	-
JWTW2	Unknown Saprolegniales	Wittwesee	-	-	-	-
JWMW1	Unknown Saprolegniales	Großer Wummsee	-	-	-	-
JWML1	Unknown Saprolegniales	Großer Wummsee	-	-	-	-
**Fungi**						
ASW11-2	Unknown fungus	Großer Stechlinsee	-	-	-	-
ASW3-23	*Cordycipitaceae*	Großer Stechlinsee	MN396371	-	MN396192	-
ASW5-1	Unknown fungus	Großer Stechlinsee	-	-	-	-
ASW11-1	*Neonectria* sp.	Großer Stechlinsee	-	-	MN396194	-
ASW5-2	Unknown fungus	Großer Stechlinsee	-	-	-	-
MSH1	*Cordycipitaceae*	Großer Stechlinsee	MN396372	-	MN396193	-
ASW9	Unknown fungus	Großer Stechlinsee	-		-	-
MSH2	*Cladosporium cladosporioides* complex	Großer Stechlinsee	MN396373	MN401001	MN396195	-
MSH3	*Cladosporium cladosporioides* complex	Großer Stechlinsee	MN396374	MN401002	MN396196	-
ASW8	*Cladosporium herbarum* complex	Großer Stechlinsee	MN396375	MN401003	MN396197	-
ASW3-22	Unknown fungus	Großer Stechlinsee	-	-	-	-
ASW1	Unknown fungus	Großer Stechlinsee	-	-	-	-
ASW4	*Phlebia radiata*	Großer Stechlinsee	MN396376	MN401004	MN396198	-
FBP1	*Penicillium brevicompactum*	Großer Stechlinsee	MN396377	MN401005	MN396199	-
FBL1	*Cladosporium herbarum* complex	Großer Stechlinsee	-	MN401006	MN396200	-
FBP3	*Penicillium brevicompactum*	Großer Stechlinsee	MN396378	MN401007	MN396201	-
FBP22	*Penicillium* sp.	Großer Stechlinsee	MN396379	-	MN396202	-
FBS1	*Didymosphaeriaceae*	Großer Stechlinsee	-	MN401008	MN396203	-
FBL3	*Heterobasidion* sp.	Großer Stechlinsee	-	MN401009	MN396204	-
FBP21	*Penicillium brevicompactum*	Großer Stechlinsee	MN396380	MN401010	MN396205	-
FBL6	Unknown fungus	Großer Stechlinsee	-	-	-	-
FBPD1	*Fusarium* sp.	Großer Stechlinsee	MN396381	MN401011	MN396206	-
FB10	*Nectriaceae*	Großer Stechlinsee	-	-	MN396207	-
FB11	*Cladosporium herbarum* complex	Großer Stechlinsee	-	MN401012	MN396208	-
FBLD2	*Cladosporium* sp.	Großer Stechlinsee	-	-	MN396209	-
FB3	*Penicillium levitum*	Großer Stechlinsee	-	-	MN396210	-
FB1	Unknown fungus	Großer Stechlinsee	-	-	-	-
FB2	Unknown fungus	Großer Stechlinsee	-	-	-	-
FB6	Unknown fungus	Großer Stechlinsee	-	-	-	-
FB7	*Cladosporium herbarum* complex	Großer Stechlinsee	-	MN401013	MN396211	-
FBP1x	*Nectria* sp.	Großer Stechlinsee	-	MN401014	MN396212	-
FBS4	Unknown fungus	Großer Stechlinsee	-	-	-	-
FBP41	*Penicillium brevicompactum*	Großer Stechlinsee	MN396382	MN401015	MN396213	-
FBP42	*Penicillium brevicompactum*	Großer Stechlinsee	-	MN401016	MN396214	-
FBP5	*Penicillium brevicompactum*	Großer Stechlinsee	-	-	MN396215	-
FBL5	*Phoma* sp.	Großer Stechlinsee	-	-	MN396216.1	-
FB12	*Cladosporium* sp.	Großer Stechlinsee	-	-	MN396217	-
FB9	*Massarina* sp.	Großer Stechlinsee	-	-	MN396218	-
FBL81	*Cladosporium cladosporioides* complex	Großer Stechlinsee	-	-	MN396219	-
FBPD2	*Cladosporium herbarum* complex	Großer Stechlinsee	-	-	MN396220	-
FBP9	*Penicillium brevicompactum*	Großer Stechlinsee	-	-	MN396221	-
FBS9	Unknown fungus	Großer Stechlinsee	-	-	-	-
FBP7	*Penicillium brevicompactum*	Großer Stechlinsee	-	-	MN396222	-
FBSL1	*Penicillium crustosum*	Großer Stechlinsee	MN396383	-	-	-
FBP8	*Cladosporium cladosporioides* complex	Großer Stechlinsee	-	MN401026	-	-
FBP81	*Penicillium brevicompactum*	Großer Stechlinsee	-	-	MN396223	-
FBP71	*Cladosporium herbarum* complex	Großer Stechlinsee	-	-	MN396224	-
FBS71	*Penicillium brevicompactum*	Großer Stechlinsee	-	-	MN396225	-
FBSS1	*Fusarium* sp.	Großer Stechlinsee	-	-	MN396226	-
FBLD2X	Unknown fungus	Großer Stechlinsee	-	-	-	-
AFL4-2	*Mortierella* sp.	Große Fuchskuhle	-	MN401032.1	-	-

## Data Availability

The data presented in this study are available on request from the corresponding author/first author.

## References

[1] Stedmon C.A., Markager S., Bro R. (2003). Tracing dissolved organic matter in aquatic environments using a new approach to fluorescence spectroscopy. Mar. Chem..

[2] Mattsson T., Kortelainen P., Räike A. (2005). Export of DOM from Boreal Catchments: Impacts of Land Use Cover and Climate. Biogeochemistry.

[3] McDonald S., Bishop A.G., Prenzler P.D., Robards K. (2004). Analytical chemistry of freshwater humic substances. Anal. Chim. Acta.

[4] Sime-Ngando T., Lefèvre E., Gleason F.H. (2011). Hidden diversity among aquatic heterotrophic flagellates: Ecological potentials of zoosporic fungi. Hydrobiologia.

[5] Romani A.M., Fischer H., Mille-Lindblom C., Tranvik L.J. (2006). Interactions of bacteria and fungi on decomposing litter: Differential extracellular enzyme activities. Ecology.

[6] Klein O.I., Isakova E.P., Deryabina Y.I., Kulikova N.A., Badun G.A., Chernysheva M.G., Stepanova E.V., Koroleva O.V. (2014). Humic Substances Enhance Growth and Respiration in the Basidiomycetes *Trametes Maxima* Under Carbon Limited Conditions. J. Chem. Ecol..

[7] Jobard M., Rasconi S., Sime-Ngando T. (2010). Diversity and functions of microscopic fungi: A missing component in pelagic food webs. Aquat. Sci..

[8] Claus H., Filip Z. (1998). Degradation and Transformation of Aquatic Humic Substances by Laccase-producing Fungi *Cladosporium cladosporioides* and *Polyporus versicolor*. Acta Hydrochim. Hydrobiol..

[9] Rojas-Jimenez K., Fonvielle J.A., Ma H., Grossart H.-P. (2017). Transformation of humic substances by the freshwater Ascomycete *Cladosporium* sp. Limnol. Oceanogr..

[10] Collado S., Oulego P., Suárez-Iglesias O., Díaz M. (2018). Biodegradation of dissolved humic substances by fungi. Appl. Microbiol. Biotechnol..

[11] van West P. (2006). *Saprolegnia parasitica*, an oomycete pathogen with a fishy appetite: New challenges for an old problem. Mycologist.

[12] Svoboda J., Mrugała A., Kozubíková-Balcarová E., Petrusek A. (2016). Hosts and transmission of the crayfish plague pathogen *Aphanomyces astaci*: A review. J. Fish Dis..

[13] Molloy D.P., Glockling S.L., Siegfried C.A., Beakes G.W., James T.Y., Mastitsky S.E., Wurdak E., Giamberini L., Gaylo M.J., Nemeth M.J. (2014). *Aquastella* gen. nov.: A new genus of saprolegniaceous oomycete rotifer parasites related to *Aphanomyces*, with unique sporangial outgrowths. Fungal Biol..

[14] Czeczuga B., Mazalska B., Godlewska A., Muszynska E. (2005). Aquatic fungi growing on dead fragments of submerged plants. Limnologica.

[15] Unestam T. (1966). Chitinolytic, Cellulolytic, and Pectinolytic Activity in vitro of Some Parasitic and Saprophytic Oomycctes. Physiol. Plant..

[16] Berner K.E., Chapman E.S. (1977). The cellulolytic activity of six Oomycetes. Mycologia.

[17] Masigol H., Khodaparast S.A., Woodhouse J.N., Rojas-Jimenez K., Fonvielle J., Rezakhani F., Mostowfizadeh-Ghalamfarsa R., Neubauer D., Goldhammer T., Grossart H. (2019). The contrasting roles of aquatic fungi and oomycetes in the degradation and transformation of polymeric organic matter. Limnol. Oceanogr..

[18] Seymour R.L. (1970). The genus *Saprolegnia*. Nova Hedwigia.

[19] Siepmann R., Johnson T.W. (1960). Isolation and culture of fungi from wood submerged in saline and fresh waters. J. Elisha Mitchell Sci. Soc..

[20] Montero-Pau J., Gomez A., Munoz J. (2008). Application of an inexpensive and high-throughput genomic DNA extraction method for the molecular ecology of zooplanktonic diapausing eggs. Limnol. Oceanogr. Methods.

[21] Borneman J., Hartin R.J. (2000). PCR Primers That Amplify Fungal rRNA Genes from Environmental Samples. Appl. Environ. Microbiol..

[22] White T., Bruns T., Lee S., Taylor J., Innis M.A., Gelfand D.H., Sininsky J.J., White T.J. (1990). Amplification and direct sequencing of fungal ribosomal RNA genes for phylogenetics. PCR Protocols: A Guide to Methods and Applications.

[23] Stielow J.B., Lévesque C.A., Seifert K.A., Meyer W., Irinyi L., Smits D., Renfurm R., Verkley G.J.M., Groenewald M., Chaduli D. (2015). One fungus, which genes? Development and assessment of universal primers for potential secondary fungal DNA barcodes. Pers. Mol. Phylogeny Evol. Fungi.

[24] Robideau G.P., De Cock A.W.A.M., Coffey M.D., Voglmayr H., Brouwer H., Bala K., Chitty D.W., Désaulniers N., Eggertson Q.A., Gachon C.M. (2011). DNA barcoding of oomycetes with cytochrome c oxidase subunit I and internal transcribed spacer. Mol. Ecol. Resour..

[25] Hall T.A. (1999). BioEdit: A user-friendly biological sequence alignment editor and analysis program for Windows 95/98/NT. Nucleic Acids Symp. Ser..

[26] Katoh K., Standley D.M. (2013). MAFFT multiple sequence alignment software version 7: Improvements in performance and usability. Mol. Biol. Evol..

[27] Kumar S., Stecher G., Tamura K. (2016). MEGA7: Molecular evolutionary genetics analysis version 7.0 for bigger datasets. Mol. Biol. Evol..

[28] Pointing S.B. (1999). Qualitative methods for the determination of lignocellulolytic enzyme production by tropical fungi. Fungal Divers..

[29] Agrawal T., Kotasthane A.S. (2012). Chitinolytic assay of indigenous Trichoderma isolates collected from different geographical locations of Chhattisgarh in Central India. SpringerPlus.

[30] Huber S.A., Balz A., Abert M., Pronk W. (2011). Characterisation of aquatic humic and non-humic matter with size-exclusion chromatography—organic carbon detection—organic nitrogen detection (LC-OCD-OND). Water Res..

[31] Paul A., Dziallas C., Zwirnmann E., Gjessing E.T., Grossart H.-P. (2011). UV irradiation of natural organic matter (NOM): Impact on organic carbon and bacteria. Aquat. Sci..

[32] Miki T., Yokokawa T., Ke P.-J., Hsieh I.-F., Hsieh C.-H., Kume T., Yoneya K., Matsui K. (2017). Statistical recipe for quantifying microbial functional diversity from EcoPlate metabolic profiling. Ecol. Res..

[33] Liu Y., Kurtán T., Wang C.Y., Lin W.H., Orfali R., Mueller W.E., Daletos G. (2016). Proksch, P. Cladosporinone, a new viridi-toxin derivative from the hypersaline lake derived fungus Cladosporium cladosporioides. J. Antibiot. Res..

[34] Zalar P.D., de Hoog G.S., Schroers H.-J., Crous P.W., Groenewald J.Z., Gunde-Cimerman N. (2007). Phylogeny and ecology of the ubiquitous saprobe *Cladosporium sphaerospermum*, with descriptions of seven new species from hypersaline environments. Stud. Mycol..

[35] Shearer C.A. (1993). The freshwater ascomycetes. Nova Hedwig..

[36] Rocha S.C., Lopez-Lastra C.C., Marano A.V., de Souza J.I., Rueda-Páramo M.E., Pires-Zottarelli C.L. (2018). New phylogenetic insights into Saprolegniales (Oomycota, Straminipila) based upon studies of specimens isolated from Brazil and Argentina. Mycol. Prog..

[37] Roberts R.E. (1963). A study of the distribution of certain members of the saprolegniales. Trans. Br. Mycol. Soc..

[38] Xu G.B., Goodell B. (2001). Mechanisms of wood degradation by brown-rot fungi: Chelator-mediated cellulose degradation and binding of iron by cellulose. J. Biotechnol..

[39] Varnaitė R., Paškevičius A., Raudonienė V. (2008). Cellulose degradation in rye straw by micromycetes and their complexes. Ekologija.

[40] Koechli C., Campbell A.N., Pepe-Ranney C., Buckley D.H. (2019). Assessing fungal contributions to cellulose degradation in soil by using high-throughput stable isotope probing. Soil Biol. Biochem..

[41] Dong Y.-C., Wang W., Hu Z.-C., Fu M.-L., Chen Q.-H. (2011). The synergistic effect on production of lignin-modifying enzymes through submerged co-cultivation of Phlebia radiata, *Dichomitus squalens* and *Ceriporiopsis subvermispora* using agricultural residues. Bioprocess Biosyst. Eng..

[42] Thompstone A., Dix N. (1985). Cellulase activity in the Saprolegniaceae. Trans. Br. Mycol. Soc..

[43] Masigol H., Khodaparast S.A., Mostowfizadeh-Ghalamfarsa R., Rojas-Jimenez K., Woodhouse J.N., Neubauer D., Grossart H.-P. (2020). Taxonomical and functional diversity of Saprolegniales in Anzali lagoon, Iran. Aquat. Ecol..

[44] Järvinen J., Taskila S., Isomäki R., Ojamo H. (2012). Screening of white-rot fungi manganese peroxidases: A comparison between the specific activities of the enzyme from different native producers. AMB Express.

[45] Liu B., Olson Å., Wu M., Broberg A., Sandgren M. (2017). Biochemical studies of two lytic polysaccharide monooxygenases from the white-rot fungus *Heterobasidion irregulare* and their roles in lignocellulose degradation. PLoS ONE.

[46] Xiao P., Mori T., Kamei I., Kondo R. (2010). A novel metabolic pathway for biodegradation of DDT by the white rot fungi, *Phlebia lindtneri* and *Phlebia brevispora*. Biogeochemistry.

[47] Piscitelli A., Giardina P., Lettera V., Pezzella C., Sannia G., Faraco V. (2011). Induction and Transcriptional Regulation of Laccases in Fungi. Curr. Genom..

[48] Feng B.Z., Li P.Q. (2012). Genome-wide identification of laccase gene family in three *Phytophthora* species. Genetica.

[49] Feng B.Z., Li P.Q. (2013). Cloning and expression of a novel laccase gene from *Phytophthora capsici*. J. Plant Pathol..

[50] Feng B.Z., Li P.Q. (2014). Cloning, characterization and expression of a novel laccase gene Pclac2 from *Phytophthora capsici*. Braz. J. Microbiol..

[51] Feng B.Z., Li P.Q., Fu L., Yu X.M. (2015). Exploring laccase genes from plant pathogen genomes: A bioinformatic approach. Genet. Mol. Res..

[52] Luis P., Gauthier A., Trouvelot S., Poinssot B., Frettinger P. (2013). Identification of Plasmopara viticola Genes Potentially Involved in Pathogenesis on Grapevine Suggests New Similarities Between Oomycetes and True Fungi. Phytopathology.

[53] Savory F., Leonard G., Richards T.A. (2015). The Role of Horizontal Gene Transfer in the Evolution of the Oomycetes. PLoS Pathog..

[54] Jiang R.H.Y., de Bruijn I., Haas B.J., Belmonte R., Löbach L., Christie J., Ackerveken G.V.D., Bottin A., Bulone V., Díaz-Moreno S.M. (2013). Distinctive Expansion of Potential Virulence Genes in the Genome of the Oomycete Fish Pathogen *Saprolegnia parasitica*. PLoS Genet..

[55] Masigol H., Khodaparast S., Mostowfizadeh-Ghalamfarsa R., Mousanejad S., Rojas-Jimenez K., Grossart H.P. (2018). Notes on Dictyuchus species (Stramenopila, Oomycetes) from Anzali lagoon, Iran. Mycol. Iran..

[56] Shi Z., Han C., Zhang X., Tian L., Wang L. (2020). Novel Synergistic Mechanism for Lignocellulose Degradation by a Thermophilic Filamentous Fungus and a Thermophilic Actinobacterium Based on Functional Proteomics. Front. Microbiol..

